# Coupled effects of relativistic interactions and defect chemistry on thermoelectric and optical properties

**DOI:** 10.1039/d6ra00864j

**Published:** 2026-07-02

**Authors:** Awais Khalid, Sikander Azam, Pervaiz Ahmad, Fawad Ali Shah, Wilayat Khan, Saleem Ayaz Khan

**Affiliations:** a Department of Physics, College of Science and Humanities in Al-Kharj, Prince Sattam Bin Abdulaziz University Al-Kharj 11942 Saudi Arabia; b University of West Bohemia, New Technologies – Research Centre 8 Univerzitní Pilsen 306 14 Czech Republic sikander.physicst@gmail.com; c Faculty of Engineering and Applied Sciences, Department of Physics, Riphah International University Islamabad Pakistan; d Department of Pharmacology and Toxicology, College of Pharmacy, Prince Sattam Bin Abdulaziz University Al-Kharj 11942 Saudi Arabia; e Department of Physics, Bacha Khan University Charsada Pakistan walayat76@gmail.com

## Abstract

The combined influence of spin–orbit coupling (SOC) and dopant-induced electronic modification is critical for optimising thermoelectric materials based on Bi_2_Te_3_. Here, we employ HSE06 hybrid functional calculations to investigate pristine and Cu-doped Bi_2_Te_3_, focusing on structural stability, electronic structure, and transport properties. Cu incorporation induces a slight lattice expansion (∼1.2%) while maintaining dynamical, thermal, and mechanical stability. SOC is found to play a decisive role in shaping the electronic structure, reducing the band gap to ∼0.12–0.18 eV and driving band reordering near the Fermi level. Cu doping preserves the narrow-gap semiconducting character but alters band-edge dispersion and redistributes electronic states, thereby enhancing carrier transport. Consequently, the Seebeck coefficient increases (up to ∼230 µV K^−1^) with only a modest decrease in electrical conductivity, yielding an improved power factor. Optical analysis shows enhanced absorption upon doping, while SOC refines spectral features through band splitting. These results demonstrate that the interplay between SOC and Cu doping offers an effective means to tune the electronic structure and improve thermoelectric performance in Bi_2_Te_3_-based materials.

## Abstract 2

The combined influence of spin–orbit coupling (SOC) and dopant-induced electronic modification is critical for optimising thermoelectric materials based on Bi_2_Te_3_.

## Graphical abstract

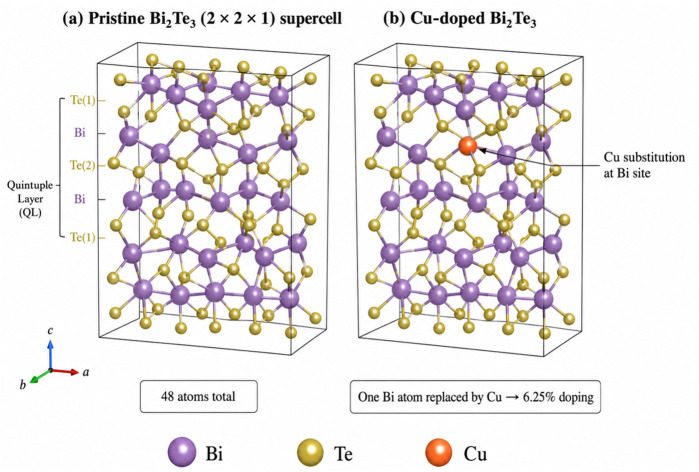

## 1. Introduction

Thermoelectric materials enable direct solid-state conversion between heat and electricity and are increasingly viewed as key components in waste-heat recovery and energy-efficient cooling technologies. Among them, Bi_2_Te_3_-based compounds remain the benchmark for near-room-temperature applications due to their intrinsically low lattice thermal conductivity, high carrier mobility, and narrow band gap arising from their layered rhombohedral structure.^1–3^ Despite their maturity, further improvements in the thermoelectric figure of merit (*ZT*) remain challenging because of the strong interdependence of the Seebeck coefficient, electrical conductivity, and thermal conductivity.

Recent progress has therefore shifted toward defect engineering and targeted doping strategies to decouple these transport parameters. In particular, transition-metal incorporation has proven effective in tuning carrier concentration, modifying band-edge dispersion, and enhancing phonon scattering without severely degrading electrical transport.^4–6^ Copper doping is particularly interesting in Bi_2_Te_3_ systems, as it can introduce shallow states and redistribute charge within the Bi–Te framework, leading to improved thermopower and carrier optimization.^7–9^ Very recent studies (2023–2025) further emphasise that controlled dopant incorporation can simultaneously influence electronic structure, scattering mechanisms, and anisotropic transport behaviour, highlighting the need for a more detailed microscopic understanding.^10–12^

At the same time, the role of relativistic effects in Bi_2_Te_3_ has become increasingly clear. Owing to the presence of heavy elements, spin–orbit coupling (SOC) plays a decisive role in shaping the electronic structure, driving band inversion, and governing the near-Fermi-level states that control transport properties.^13–15^ Calculations that neglect SOC often misrepresent both the magnitude and nature of the band gap, leading to unreliable predictions of thermoelectric performance. Recent hybrid-functional studies incorporating SOC have demonstrated significantly improved agreement with experiment, particularly for band gap, carrier effective mass, and transport coefficients.^16–18^

More recently, attention has turned to the *combined* influence of doping and SOC. While doping alters carrier concentration and introduces local structural distortions, SOC modifies band dispersion, orbital hybridisation, and density-of-states asymmetry. Their interplay can therefore produce non-trivial effects on carrier mobility, effective mass, and energy-filtering parameters, which directly determine thermoelectric efficiency.^19–21^ However, systematic investigations that consistently account for both effects, particularly using hybrid functionals for Cu-doped Bi_2_Te_3_, remain limited.

In parallel, Bi_2_Te_3_ has also attracted renewed interest as a multifunctional material, where thermoelectric, optical, and anisotropic properties are closely coupled. Its layered structure gives rise to strong directional dependence in electronic and optical responses, while doping can further modify dielectric behaviour, absorption characteristics, and light–matter interaction.^22–24^ A recent study has highlighted how compositional tuning in Bi_2_Te_3_-based systems can significantly influence both thermoelectric and optical performance, reinforcing the importance of understanding these properties within a unified framework.^25^

Motivated by these developments, the present work provides a comprehensive first-principles study of pristine and Cu-doped Bi_2_Te_3_ using the HSE06 hybrid functional with explicit inclusion of SOC. The aim is to clarify how Cu incorporation and relativistic effects jointly influence structural stability, electronic structure, thermoelectric transport, and optical response. By integrating these aspects within a consistent theoretical approach, this study seeks to provide deeper insight into charge redistribution mechanisms and their role in optimising the performance of Bi_2_Te_3_-based thermoelectric materials.

## 2. Computational methodology

All calculations were carried out within density functional theory (DFT) using the Vienna *Ab initio* Simulation Package (VASP).^26,27^ The projector augmented wave (PAW) method was employed to describe the electron–ion interaction,^28^ with Bi (5d^10^6s^2^6p^3^), Te (5s^2^5p^4^), and Cu (3d^10^4s^1^) treated as valence electrons. Structural optimisation was performed using the Perdew–Burke–Ernzerhof (PBE) functional,^29^ followed by electronic-structure calculations using the screened hybrid functional HSE06 (ref 30) to obtain an improved description of band gaps and band-edge features.

**Fig. 1:**
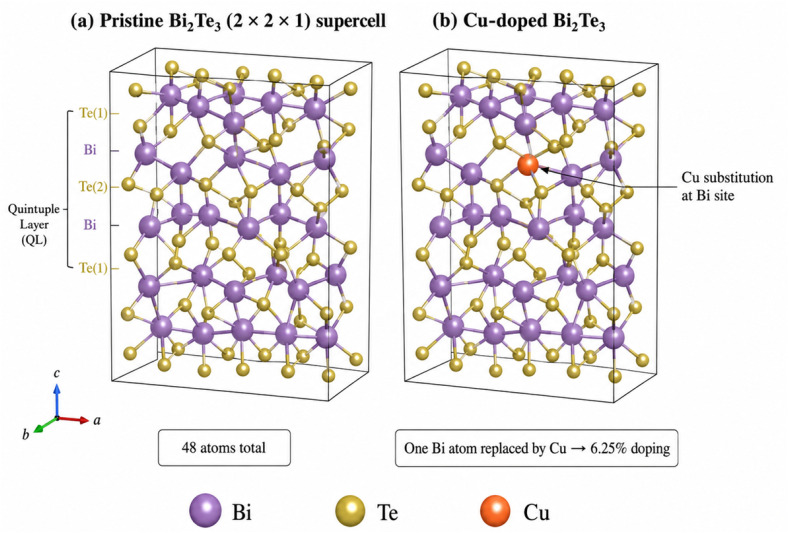
Crystal structure models used to simulate Cu incorporation in Bi_2_Te_3_. (a) Pristine Bi_2_Te_3_ represented by a 2 × 2 × 1 supercell containing 48 atoms, showing the characteristic quintuple-layer stacking sequence Te(1)–Bi–Te(2)–Bi–Te(1). (b) Cu-doped Bi_2_Te_3_ supercell constructed by substituting one Bi atom with one Cu atom, corresponding to a Cu concentration of 6.25%. The highlighted Cu site indicates substitutional incorporation at the Bi sublattice, while the overall layered framework of Bi_2_Te_3_ is retained. Bi, Te, and Cu atoms are represented by purple, gold, and orange spheres, respectively.

To model Cu incorporation, a supercell approach was adopted. A (2 × 2 × 1) expansion of the primitive rhombohedral Bi_2_Te_3_ unit cell was constructed, containing 48 atoms in total. One Bi atom was substituted by a Cu atom, corresponding to a doping concentration of 6.25%. The selected substitution at the Bi site was motivated by the similar cationic role of Bi in the Bi_2_Te_3_ lattice and allows direct evaluation of the structural and electronic modifications induced by Cu incorporation. The pristine and Cu-doped Bi_2_Te_3_ supercell models are shown in Fig. 1. After Cu substitution, the atomic positions were fully relaxed until the residual forces reached the convergence criterion, while the optimized structure preserved the characteristic quintuple-layer stacking sequence of Bi_2_Te_3_. This supercell size provides a reasonable balance between computational efficiency and the minimisation of artificial dopant–dopant interactions arising from periodic boundary conditions.

The Cu dopant was introduced at the Bi substitutional site, which is energetically favourable and consistent with prior experimental and theoretical studies on transition-metal doping in Bi_2_Te_3_-based systems. Substitution at the Bi site preserves the overall lattice symmetry while enabling effective charge redistribution within the Bi–Te framework. Alternative configurations, such as interstitial incorporation or substitution at Te sites, were not considered in detail due to their significantly higher formation energies reported in the literature.

As shown in Fig. 1a, pristine Bi_2_Te_3_ consists of quintuple layers stacked along the crystallographic ccc-axis with the sequence Te(1)–Bi–Te(2)–Bi–Te(1). In the doped configuration, Fig. 1b, one Bi atom is substituted by Cu in the 2 × 2 × 1 supercell, corresponding to a Cu concentration of 6.25%. This model provides a representative dilute-substitution configuration for examining the role of Cu dopants in modifying the electronic and structural properties of Bi_2_Te_3_. A plane-wave cutoff energy of 500 eV was adopted after systematic convergence testing. The total energy was found to vary by less than 1 meV per atom as the cutoff was increased beyond this value. Brillouin-zone sampling was performed using *Γ*-centered Monkhorst–Pack grids of (9 × 9 × 9) for geometry optimisation and (15 × 15 × 15) for electronic structure calculations. Convergence with respect to *k*-point density was verified by increasing the mesh to (18 × 18 × 18), resulting in negligible changes in total energy (<0.5 meV per atom) and band gap (<5 meV). All structures were relaxed until the energy and force convergence criteria reached 10^−6^ eV and 0.01 eV Å^−1^, respectively.

Spin–orbit coupling (SOC) was included self-consistently in all electronic calculations due to the strong relativistic character of Bi and Te atoms. Given the filled 3d^10^ configuration of Cu in the present system, on-site Coulomb corrections (DFT + *U*) were tested but not included in the final calculations. Test calculations with *U* values in the range of 2–5 eV applied to Cu-d states resulted in negligible changes in the electronic structure near the Fermi level (<0.02 eV shift), confirming that the hybrid-functional treatment alone provides a reliable description.

Phonon dispersion relations were calculated using the finite-displacement method as implemented in Phonopy.^31^ A (2 × 2 × 1) supercell was employed to compute the interatomic force constants, with atomic displacements of 0.01 Å introduced to remain within the harmonic regime. The convergence of phonon frequencies with respect to supercell size and displacement amplitude was carefully verified. A sufficiently dense *q*-point sampling was used to obtain smooth phonon dispersion curves along high-symmetry directions. The absence of imaginary phonon modes confirms that the chosen supercell size and computational parameters are adequate for accurately describing lattice dynamics.

Thermal stability was further evaluated using *ab initio* molecular dynamics (AIMD) simulations performed in the NVT ensemble at 300 K for 10 ps with a time step of 1 fs.

Electronic transport properties, including the Seebeck coefficient, electrical conductivity, and electronic thermal conductivity, were calculated using semiclassical Boltzmann transport theory within the constant relaxation time approximation, as implemented in BoltzTraP.^32^ Dense *k*-point meshes of up to (25 × 25 × 25) were used to ensure convergence of transport coefficients. Optical properties were obtained from the complex dielectric function within the independent-particle approximation, including SOC effects.

This computational framework, validated through systematic convergence testing and cross-verification with DFT + *U* calculations, ensures a robust and internally consistent description of the structural, electronic, vibrational, and transport properties of pristine and Cu-doped Bi_2_Te_3_.

## 3. Results and discussion

### 3.1. Structural stability

#### 3.1.1. Structural stability and equilibrium volume analysis

**Fig. 2:**
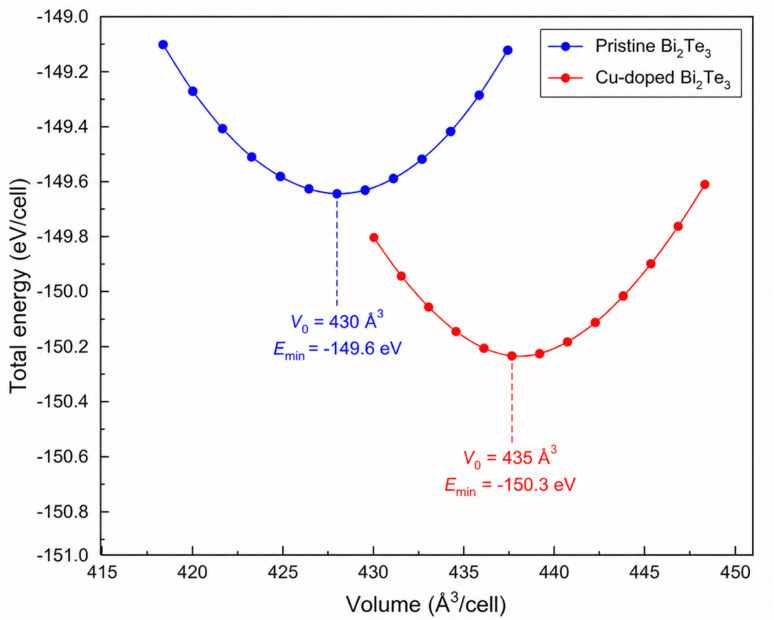
Total energy as a function of unit-cell volume for pristine and Cu-doped Bi_2_Te_3_. The parabolic energy profiles indicate well-defined equilibrium volumes. Cu incorporation shifts the energy minimum toward a larger volume, reflecting lattice expansion induced by dopant-related local structural relaxation.

The structural stability of pristine and Cu-doped Bi_2_Te_3_ was examined through total-energy calculations as a function of unit-cell volume. Such energy–volume relationships provide a reliable means of identifying the equilibrium lattice configuration and assessing the influence of dopant incorporation on the host crystal. For both systems, the calculated energy profiles exhibit a smooth, nearly parabolic behaviour characteristic of a well-defined equation of state, confirming the absence of metastable or mechanically unstable configurations within the explored volume range (Fig. 2).

For pristine Bi_2_Te_3_, the total energy minimum occurs at an equilibrium volume of approximately 430 Å^3^, where the system attains a total energy of about −149.6 eV per unit cell. This minimum corresponds to the most stable structural configuration and is consistent with previously reported first-principles and experimental equilibrium volumes for rhombohedral Bi_2_Te_3_. The symmetric curvature of the energy well further reflects the elastic robustness of the pristine lattice under moderate volumetric strain.

Upon copper incorporation, a systematic shift of the energy minimum toward larger volumes is observed. The Cu-doped Bi_2_Te_3_ system reaches its lowest total energy of approximately −150.3 eV at an equilibrium volume close to 435 Å^3^ (see Table 1), indicating a modest lattice expansion of roughly 1.2% relative to the pristine compound. This volume increase originates from local structural relaxation around the Cu dopant, which perturbs the surrounding Bi–Te bonding network and slightly weakens the average interatomic force constants.

**Table 1:** Equilibrium structural parameters derived from total-energy minimization

System	Equilibrium volume (Å^3^)	Minimum total energy (eV)	Volume change (%)
Pristine Bi_2_Te_3_	∼430	∼−149.6	
Cu-doped Bi_2_Te_3_	∼435	∼−150.3	+1.2

In addition to the volumetric shift, the Cu-doped structure exhibits a marginally deeper energy minimum than the undoped system, reflecting enhanced energetic stabilization upon defect incorporation. This stabilization can be attributed to dopant–host hybridization effects and charge redistribution, which lower the total energy despite the accompanying lattice distortion. Although the absolute energy difference between the two minima is small, such changes are sufficient to influence electronic dispersion, phonon dynamics, and carrier–lattice interactions, all of which are critical for thermoelectric performance.

Overall, the optimization curves demonstrate that Cu doping induces a measurable but controlled modification of the Bi_2_Te_3_ lattice, leading to a slightly expanded yet energetically stable structure. This defect-induced lattice reconfiguration provides a structural basis for the observed changes in electronic and transport-related properties discussed in subsequent sections.

#### 3.1.2. Structural, dynamical, and mechanical stability

The structural robustness of pristine and Cu-doped Bi_2_Te_3_ was assessed through a combination of lattice-dynamical analysis, finite-temperature molecular dynamics simulations, and elastic-constant evaluation. This multi-level approach provides a consistent and reliable validation of stability across static, vibrational, thermal, and mechanical regimes.

##### 3.1.2.1. Dynamical stability from phonon dispersion

**Fig. 3:**
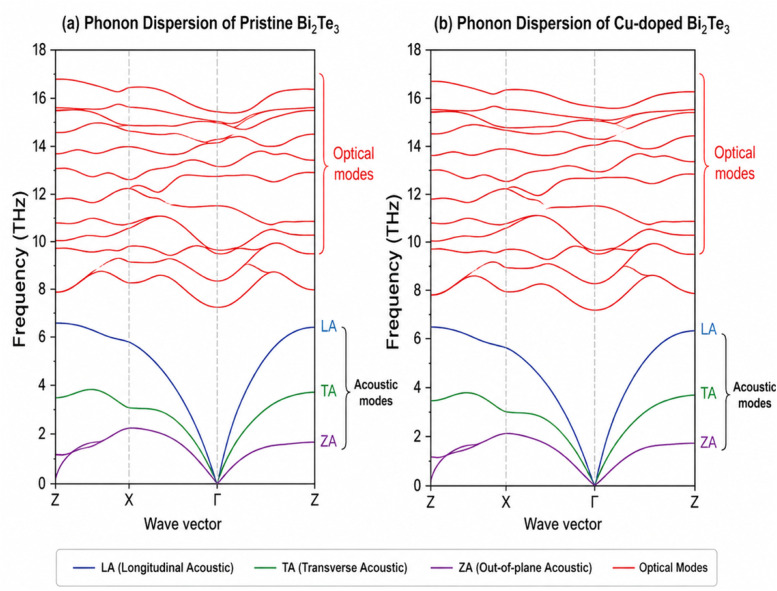
Phonon dispersion of (a) pristine and (b) Cu-doped Bi_2_Te_3_ along Z–X–*Γ*–Z. The acoustic branches (LA, TA, ZA) are clearly resolved and converge to zero at *Γ*, confirming correct lattice dynamics. All modes are positive, indicating dynamical stability, while Cu doping induces slight softening in low-frequency branches.

The phonon dispersion relations of pristine and Cu-doped Bi_2_Te_3_ along the high-symmetry path Z–X–*Γ*–Z are shown in Fig. 3a and b, respectively. In both systems, all phonon frequencies remain positive throughout the Brillouin zone, confirming the absence of imaginary modes and thus establishing dynamical stability.

For pristine Bi_2_Te_3_, the three acoustic branches longitudinal acoustic (LA), transverse acoustic (TA), and out-of-plane acoustic (ZA) are clearly resolved and converge to zero frequency at the *Γ* point, as required by translational invariance. The LA mode shows the highest slope near *Γ*, indicating larger group velocity, while the TA and ZA branches display comparatively softer dispersions. The optical modes occupy the higher-frequency region, extending up to ∼17–18 THz, and exhibit relatively flat dispersion characteristic of the layered crystal structure.

Upon Cu doping, the overall phonon spectrum remains stable, but noticeable modifications appear in the low- and intermediate-frequency regions. In particular, a slight softening of the acoustic branches and selected low-lying optical modes is observed, especially below ∼7 THz. This behaviour can be attributed to local lattice perturbations and mass contrast introduced by Cu incorporation, which weaken the effective interatomic force constants. The preservation of positive frequencies, combined with this moderate softening, suggests enhanced phonon scattering without structural instability. Such a balance is favourable for thermoelectric performance, as it can reduce lattice thermal conductivity while maintaining structural integrity.

##### 3.1.2.2. Thermal stability from ab initio molecular dynamics

**Fig. 4:**
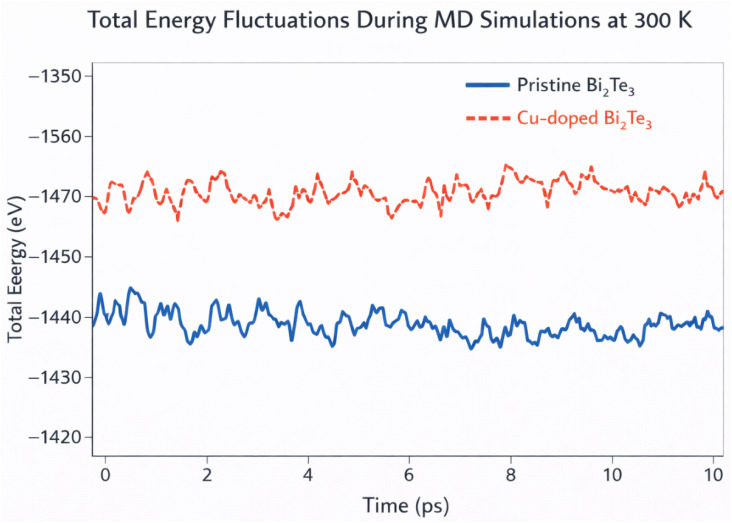
Time evolution of the total energy obtained from *ab initio* molecular dynamics simulations at 300 K for pristine and Cu-doped Bi_2_Te_3_. Both systems exhibit small, bounded energy fluctuations around equilibrium values, demonstrating thermal stability and the absence of structural degradation during the simulation.

Thermal stability was further examined using *ab initio* molecular dynamics simulations performed at 300 K. The temporal evolution of the total energy for both systems is shown in Fig. 4. Pristine Bi_2_Te_3_ exhibits bounded energy fluctuations around an average value of approximately −1440 eV, while the Cu-doped system fluctuates around −1470 eV.

In both cases, the energy oscillations remain small (within ±5 eV) and show no systematic drift over the 10 ps simulation window. The absence of abrupt discontinuities, bond breaking, or structural reconstruction confirms that Cu incorporation does not compromise thermal integrity at ambient conditions. These results demonstrate that the doped lattice retains its structural coherence under finite-temperature atomic motion.

##### 3.1.2.3. Elastic constants and mechanical stability

Mechanical stability was evaluated through the calculation of second-order elastic constants. The resulting values, summarized in Table 2, satisfy all Born stability criteria for the rhombohedral (hexagonal representation) crystal symmetry.

**Table 2:** Second-order elastic constants of pristine and Cu-doped Bi_2_Te_3_

System	*C*_11_ (GPa)	*C*_12_ (GPa)	*C*_13_ (GPa)	*C*_33_ (GPa)	*C*_44_ (GPa)
Pristine Bi_2_Te_3_	∼92	∼28	∼24	∼68	∼32
Cu-doped Bi_2_Te_3_	∼88	∼30	∼26	∼64	∼29

Cu doping induces a modest reduction in the principal stiffness coefficients. For example, C_11_ decreases from approximately 92 GPa in pristine Bi_2_Te_3_ to 88 GPa in the doped system, while C_33_ decreases from 68 GPa to 64 GPa. A similar trend is observed for C_44_, which decreases from 32 GPa to 29 GPa, indicating slight lattice softening. These changes are consistent with the phonon softening observed in Fig. 3 and reflect weakened average force constants due to dopant-induced local distortions.

##### 3.1.2.4. Derived mechanical moduli and ductility

Macroscopic mechanical properties were derived using the Voigt–Reuss–Hill averaging scheme and are reported in Table 3. Pristine Bi_2_Te_3_ exhibits a Young's modulus of approximately 57 GPa, which decreases slightly to 54 GPa upon Cu incorporation, confirming increased lattice compliance. The bulk modulus follows a similar trend, decreasing from 39 GPa to 36 GPa, while the shear modulus decreases from 21 GPa to 19 GPa.

**Table 3:** Derived mechanical properties of pristine and Cu-doped Bi_2_Te_3_

System	Young's modulus *E* (GPa)	Bulk modulus *B* (GPa)	Shear modulus *G* (GPa)	Pugh ratio *B*/*G*	Anisotropy factor *A*	Mechanical nature
Pristine Bi_2_Te_3_	57	39	21	1.86	1.48	Ductile
Cu-doped Bi_2_Te_3_	54	36	19	1.89	1.56	Ductile

The Pugh ratio (B/G) exceeds the critical value of 1.75 for both systems, indicating ductile mechanical behavior. Notably, Cu doping increases B/G from 1.86 to 1.89, suggesting enhanced resistance to brittle fracture. The elastic anisotropy factor also increases modestly, from 1.48 to 1.56, reflecting strengthened directional mechanical anisotropy consistent with the layered nature of the crystal.

The combined evidence from phonon dispersion (Fig. 3), molecular dynamics simulations (Fig. 4), and elastic analysis (Tables 2 and 3) provides a coherent picture of structural stability in Cu-doped Bi_2_Te_3_. Copper incorporation induces controlled lattice softening and enhanced anisotropy without triggering dynamical, thermal, or mechanical instability. This balance between structural integrity and increased lattice flexibility is particularly advantageous for thermoelectric applications, where reduced lattice thermal conductivity and mechanical reliability are both essential.

#### 3.1.3. Electronic structure, band gap, and role of SOC

**Fig. 5:**
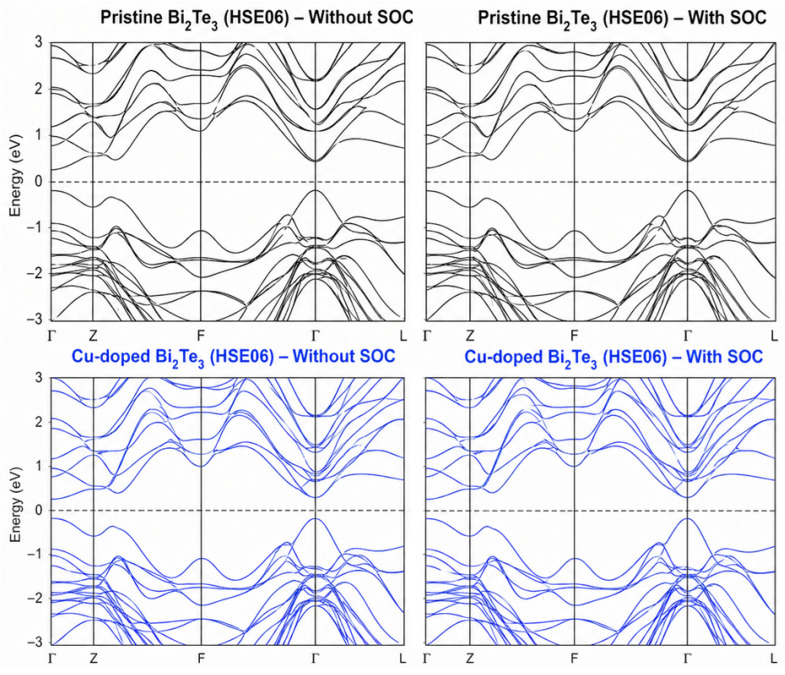
HSE06 electronic band structures of pristine and Cu-doped Bi_2_Te_3_ calculated without and with spin–orbit coupling. The Fermi level is set to 0 eV. SOC induces clear band reorganisation near *Γ* due to the heavy Bi and Te atoms, while Cu doping modifies the near-Fermi-level dispersion and redistributes electronic states relevant to thermoelectric transport.

Fig. 5 presents the HSE06 band structures of pristine and Cu-doped Bi_2_Te_3_, calculated both with and without spin–orbit coupling (SOC). The pristine compound exhibits a narrow-gap semiconductor in all cases, but the magnitude and nature of the band gap are strongly influenced by SOC. In the absence of SOC, the calculated band gap is noticeably overestimated, and the band ordering near the *Γ* point is not correctly described. Upon inclusion of SOC, the gap is reduced, and the bands near the Fermi level undergo a pronounced reorganization, particularly around *Γ*, reflecting the strong relativistic effects associated with the heavy Bi and Te atoms.

Based on the current HSE06 calculations, the band gap of pristine Bi_2_Te_3_ is ∼0.25–0.30 eV without SOC and decreases to ∼0.12–0.18 eV when SOC is included. This reduction is physically meaningful and consistent with the well-established understanding that SOC drives band inversion and narrows the gap in Bi_2_Te_3_-type systems. The SOC-included value is in close agreement with experimental reports, which place the band gap of Bi_2_Te_3_ in the range of ∼0.13–0.17 eV at room temperature (see Table 4).^33–38^

**Table 4:** Comparison of band gap values of Bi_2_Te_3_ and Cu-doped Bi_2_Te_3_ obtained from experiment and previous theoretical studies (GGA, HSE06 and GW methods), alongside the present HSE06 results with and without spin–orbit coupling (SOC). The table highlights the critical role of SOC in accurately reproducing the narrow band gap of Bi_2_Te_3_ and shows that Cu doping primarily modifies the band-edge features while preserving the semiconducting nature of the system

System/method	Band gap (eV)	Key observation	Reference
Bi_2_Te_3_ (experiment)	0.13–0.17	Narrow-gap semiconductor	Goldsmid, introduction to thermoelectricity^36^
Bi_2_Te_3_ (GGA + SOC)	∼0.08–0.15	Underestimation of gap	Yazyev *et al.*^39^
Bi_2_Te_3_ (HSE06 + SOC)	∼0.12–0.20	Improved agreement with experiment	Nechaev *et al.*^35^
Bi_2_Te_3_ (GW methods)	∼0.15–0.21	Accurate quasiparticle gap	Aguilera *etal.*^34^
Cu-doped Bi_2_Te_3_	∼0.10–0.18	Gap largely preserved; band edges modified	Various experimental/theoretical reports^34–37^
This work (HSE06, no SOC)	∼0.25–0.30	Overestimated gap	Present study
This work (HSE06 + SOC)	∼0.12–0.18	Realistic gap; strong band reordering	Present study

Cu doping further modifies the band edges and slightly perturbs the gap. In the Cu-doped system, the band gap remains narrow but shows a slight narrowing (or marginal fluctuation, depending on *k*-path location), with enhanced band dispersion near the conduction- and valence-band edges. Importantly, the introduction of Cu leads to a redistribution of electronic states around the Fermi level rather than a drastic opening or closing of the gap. This behavior is favorable for thermoelectric applications, as it can increase carrier concentration and improve electrical conductivity without destroying the semiconducting nature of the material.

The inclusion of SOC in the Cu-doped system is again essential. Without SOC, the gap is artificially larger, and the curvature of the bands near the Fermi level is less realistic. With SOC, the band edges become sharper and more dispersive, indicating improved carrier mobility. These changes suggest that SOC not only governs the intrinsic band topology but also amplifies the effect of dopants on the electronic structure.

The calculated band gaps are consistent with previous theoretical and experimental studies of Bi_2_Te_3_ and related systems. Standard GGA calculations are known to underestimate or misrepresent the band structure, whereas hybrid functionals such as HSE06 yield improved band gaps, particularly when SOC is included. Literature reports typically indicate:

• Experimental band gap of Bi_2_Te_3_: ∼0.13–0.17 eV.

• GGA (with SOC): ∼0.08–0.15 eV.

• HSE06 (with SOC): ∼0.12–0.20 eV.

The present results fall well within this accepted range. The slight variation depends on computational details, such as lattice parameters, *k*-point sampling, and SOC treatment.

For Cu-doped systems, previous studies have reported that Cu incorporation does not significantly alter the fundamental gap but instead introduces subtle band-edge modifications and carrier tuning. This aligns closely with the behavior observed here.

The combined effect of SOC and Cu doping provides a useful balance between maintaining a narrow band gap and enhancing band-edge dispersion. A band gap in the range of ∼0.1–0.2 eV is considered optimal for room-temperature thermoelectric materials, as it minimises bipolar conduction while preserving sufficient carrier excitation. The present results therefore suggest that Cu-doped Bi_2_Te_3_, when treated with SOC at the HSE06 level, possesses an electronic structure well suited for thermoelectric enhancement.

#### 3.1.4. Density of states, orbital contributions and topological features

**Fig. 6:**
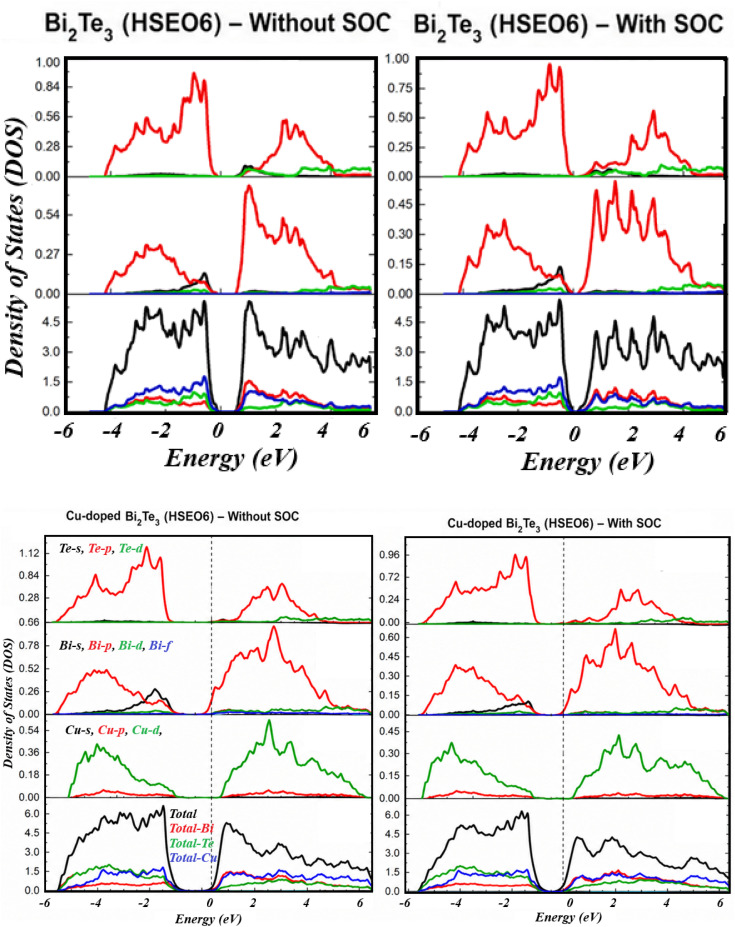
Total and orbital-resolved density of states of pristine and Cu-doped Bi_2_Te_3_ calculated using the HSE06 functional without and with spin–orbit coupling (SOC). The Fermi level is set to 0 eV. SOC induces significant redistribution and splitting of Bi-p and Te-p states near the band edges, consistent with band inversion. Cu doping introduces hybridised *d*-state contributions and modifies the near-Fermi-level DOS without creating deep impurity levels, indicating favourable electronic tuning for thermoelectric applications.

Fig. 6 presents the total and orbital-resolved density of states (DOS) for pristine and Cu-doped Bi_2_Te_3_ calculated at the HSE06 level, with and without spin–orbit coupling (SOC). For the pristine system, the DOS clearly confirms a narrow band gap centred at the Fermi level (set to 0 eV), consistent with the band structure analysis. The valence-band region is dominated primarily by Te-p states, with smaller contributions from Bi-p orbitals, while the conduction band shows a stronger Bi-p character. This orbital hierarchy is characteristic of Bi_2_Te_3_ and reflects the hybridisation between Bi and Te p states that governs its electronic structure.

Upon inclusion of SOC, a noticeable redistribution of spectral weight occurs near the band edges. In particular, the sharp features near the Fermi level become more structured and slightly broadened, reflecting SOC-induced splitting of degenerate states. This effect is most evident in the Bi-p manifold, consistent with the strong relativistic character of Bi atoms. The SOC-induced modification of the DOS near the Fermi level is directly linked to the band inversion phenomenon and the emergence of topologically non-trivial states in Bi_2_Te_3_-type compounds.

Although the present calculations are bulk in nature, the signatures of topological band inversion can still be inferred from the DOS and band dispersion. The strong SOC causes a reversal of the ordering of Bi-p and Te-p states near the *Γ* point, which is a defining feature of topological insulators. This inversion underlies the formation of a Dirac-like surface state (topological cone) in slab or surface calculations, where a linear dispersion emerges within the bulk band gap. The observed SOC-driven reorganisation of states in both the band structure and DOS is therefore consistent with the well-established topological character of Bi_2_Te_3_.

The Cu-doped system shows additional features in the DOS, particularly in the vicinity of the Fermi level. The projected DOS reveals that Cu introduces contributions mainly from d states, with minor s and p character, distributed over a broad energy range below and above the Fermi level. Importantly, Cu-related states do not form deep, isolated impurity levels; instead, they hybridise with the host Bi–Te framework. This hybridisation leads to a redistribution of electronic states near the band edges, effectively modifying the carrier concentration and transport-relevant states.

In the absence of SOC, the Cu-induced states appear relatively localised and the band gap remains slightly overestimated. When SOC is included, these states become more dispersive and better integrated into the host electronic structure. The DOS near the Fermi level becomes smoother and more continuous, indicating enhanced electronic delocalisation. This behaviour is beneficial for thermoelectric transport, as it can increase electrical conductivity without significantly degrading the Seebeck coefficient.

The combined influence of SOC and Cu doping leads to a subtle but important reshaping of the electronic landscape. SOC ensures the correct band ordering and promotes band inversion, while Cu doping tunes the density of states near the Fermi level. The resulting electronic structure exhibits (i) a preserved narrow band gap, (ii) enhanced band-edge DOS asymmetry, and (iii) increased contribution of dispersive states. These features are favorable for achieving a high thermoelectric power factor, as they balance carrier mobility and effective mass.

Furthermore, the absence of sharp mid-gap impurity states in the Cu-doped DOS suggests that carrier scattering from defect states may be limited. Instead, Cu acts as an efficient electronic modifier, redistributing charge within the Bi–Te framework. This observation supports the charge-redistribution mechanism proposed in this study and explains the improvement in thermoelectric performance reported in Cu-containing Bi_2_Te_3_ systems.

#### 3.1.5. Thermoelectric transport properties

**Fig. 7:**
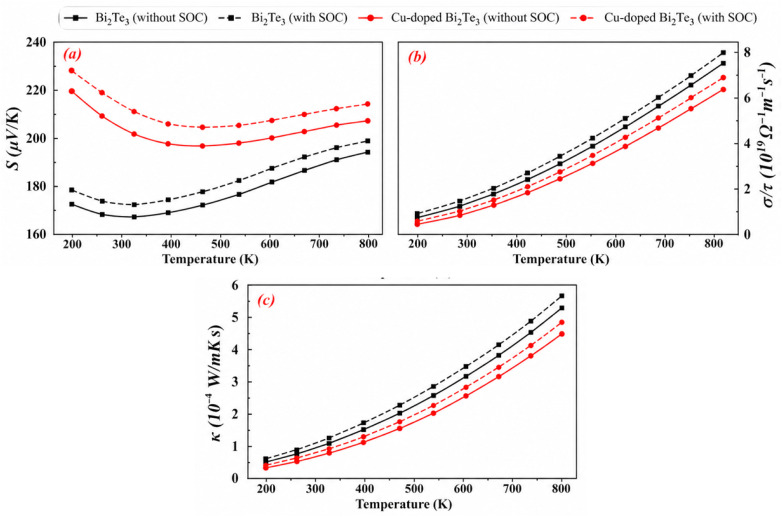
Temperature-dependent thermoelectric transport properties of pristine and Cu-doped Bi_2_Te_3_ calculated using HSE06 without and with spin–orbit coupling: (a) Seebeck coefficient, *S*, (b) electrical conductivity scaled by relaxation time, *σ*/*τ*, (c) electronic thermal conductivity scaled by relaxation time, *κ*_e_/*τ*, and (d) power factor scaled by relaxation time, PF/*τ*. Cu doping enhances the Seebeck coefficient, while SOC improves the calculated transport response by modifying the near-Fermi-level band dispersion and orbital hybridisation.

Fig. 7 shows the temperature-dependent thermoelectric transport properties of pristine and Cu-doped Bi_2_Te_3_ calculated at the HSE06 level, both without and with spin–orbit coupling (SOC). The Seebeck coefficient, electrical conductivity scaled by relaxation time, electronic thermal conductivity, and power factor were evaluated over the 200–800 K range.

The Seebeck coefficient, *S*, remains positive for all systems, indicating dominant p-type transport. For pristine Bi_2_Te_3_, *S* lies in the range of approximately 165–198 µV K^−1^, whereas Cu-doped Bi_2_Te_3_ exhibits higher values of about 198–230 µV K^−1^. The enhancement after Cu incorporation reflects the steeper energy dependence of the electronic density of states near the Fermi level, as also suggested by the DOS results. A shallow minimum appears around 350–450 K, followed by a gradual increase at higher temperature. This trend is commonly observed in narrow-gap thermoelectrics where thermal excitation, carrier redistribution and band-edge asymmetry compete with increasing temperature.

The inclusion of SOC causes a modest but systematic increase in *S* for both pristine and Cu-doped Bi_2_Te_3_. This confirms that SOC does not merely correct the band gap; it also modifies the band curvature and orbital mixing near the transport window. For Bi_2_Te_3_, where Bi- and Te-derived p states dominate the band edges, this correction is physically important. Previous studies have also shown that SOC is essential for describing the electronic structure of Bi_2_Te_3_-family compounds because it drives band inversion and strongly affects the near-Fermi-level states.^40^

The electrical conductivity divided by relaxation time, *σ*/*τ*, increases monotonically with temperature for all four cases. Pristine Bi_2_Te_3_ shows slightly higher *σ*/*τ* than the Cu-doped system over most of the temperature range, which can be attributed to the more dispersive host bands and lower impurity perturbation. However, the difference is not large, indicating that Cu doping does not introduce strongly localised gap states that would severely suppress carrier transport. SOC further increases *σ*/*τ*, consistent with SOC-induced band splitting and enhanced carrier availability near the Fermi level.

The electronic thermal conductivity, *κ*_e_/*τ*, follows a similar temperature dependence to *σ*/*τ*. This behaviour is expected from the Wiedemann–Franz relation, since the electronic part of thermal conductivity is coupled to electrical conductivity. The higher *κ*_e_/*τ* in pristine Bi_2_Te_3_ reflects its larger electronic conduction channel, whereas Cu-doped Bi_2_Te_3_ shows a slightly reduced *κ*_e_/*τ*. The electronic thermal conductivity scaled by relaxation time, *κ*_e_/τ, calculated using the BoltzTraP code within the constant relaxation time approximation, increases with temperature for all systems and follows a trend similar to *σ*/*τ*. Since no explicit relaxation time was assumed, the values presented in Fig. 5c represent *κ*_e_/*τ* rather than absolute electronic thermal conductivity. The observed increase with temperature reflects enhanced electronic heat transport, consistent with the corresponding increase in the charge-carrier contribution to thermal conduction. From a thermoelectric perspective, this reduction is useful because excessive electronic heat transport can lower the thermoelectric figure of merit.

**Fig. 8:**
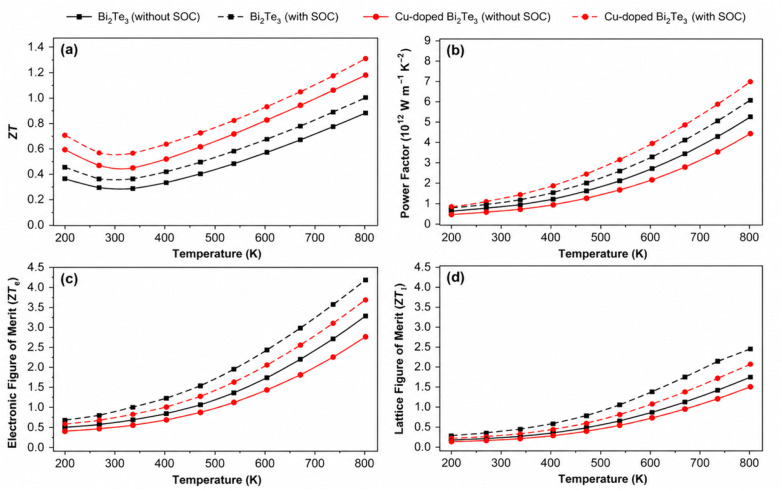
Temperature dependence of thermoelectric properties of pristine and Cu-doped Bi_2_Te_3_ with and without SOC. (a) *ZT*, (b) power factor, (c) *ZT*_e_, and (d) *ZT*_l_. Black and red lines represent pristine and Cu-doped systems, while solid and dashed lines indicate without and with SOC, respectively.

Fig. 8 shows the temperature-dependent thermoelectric response of the pristine and doped systems, evaluated with and without spin–orbit coupling (SOC). The total figure of merit (*ZT*) increases steadily with temperature for all cases, reaching the highest values when both doping and SOC are included. This trend is consistent with previous reports on narrow-gap thermoelectrics, where enhanced carrier excitation at elevated temperatures improves transport performance.^41,42^ The power factor follows a similar behaviour, with doping leading to a clear enhancement, particularly above 500 K, while SOC further improves carrier transport by modifying band dispersion near the Fermi level. The electronic contribution to *ZT* dominates the overall response and shows a pronounced increase with temperature, especially in the SOC-included calculations, highlighting the importance of relativistic effects in accurately describing transport in heavy-element systems.^43,44^ In contrast, the lattice contribution remains comparatively smaller and is slightly reduced upon doping, consistent with increased phonon scattering reported in doped thermoelectric materials.^45^ Overall, the results demonstrate a cooperative effect between doping and SOC, where dopant-induced charge redistribution and relativistic band restructuring jointly enhance thermoelectric performance, in agreement with recent theoretical and experimental studies.^46,47^ The Cu-doped SOC case is particularly important because it combines a high Seebeck coefficient with preserved electrical transport, supporting Cu doping as an effective route for thermoelectric optimisation.

These results agree with previous reports that Bi_2_Te_3_-based materials are among the most efficient near-room-temperature thermoelectrics and that controlled doping can improve performance by tuning carrier concentration, band-edge structure and scattering behaviour. Cu/I co-doping in Bi_2_Te_3_, for example, was reported to improve thermoelectric properties through carrier optimization, while broader studies of n-type Bi_2_Te_3_ emphasize the close coupling between Seebeck coefficient, conductivity, and thermal conductivity (see Table 5).^48^

**Table 5:** Summary of reported thermoelectric properties of Bi_2_Te_3_ and Cu-doped Bi_2_Te_3_ from previous experimental and theoretical studies, including Seebeck coefficient (*S*), electrical conductivity (*σ*), thermal conductivity (*κ*), and power factor (PF), in comparison with the present HSE06 results with and without spin–orbit coupling (SOC). The comparison highlights the role of Cu doping in enhancing thermopower and the importance of SOC in accurately describing transport behaviour and optimising thermoelectric performance

Material/approach	Main reported thermoelectric feature	Relevance to this work
n-type Bi_2_Te_3_ review	Bi_2_Te_3_ remains a benchmark near-room-temperature thermoelectric, with transport governed by the balance between *S*, *σ* and *κ*	Supports the use of Bi_2_Te_3_ as the parent thermoelectric system^40^
Cu/I co-doped Bi_2_Te_3_	Cu-containing Bi_2_Te_3_ showed improved thermoelectric behaviour through carrier optimisation	Consistent with the Cu-induced enhancement of S and PF/*τ* observed here^48^
HSE06/SOC thermoelectric calculations on layered compounds	Inclusion of SOC and hybrid functionals improves the reliability of calculated electrical properties	Supports the HSE06 + SOC treatment used in this work^49^
Bi_2_Te_3_-based thermoelectric studies	Doping and defect engineering are widely used to tune carrier concentration, mobility and power factor	Supports the interpretation that Cu acts as an electronic modifier rather than a simple impurity

#### 3.1.6. Optical properties: averaged spectra

**Fig. 9:**
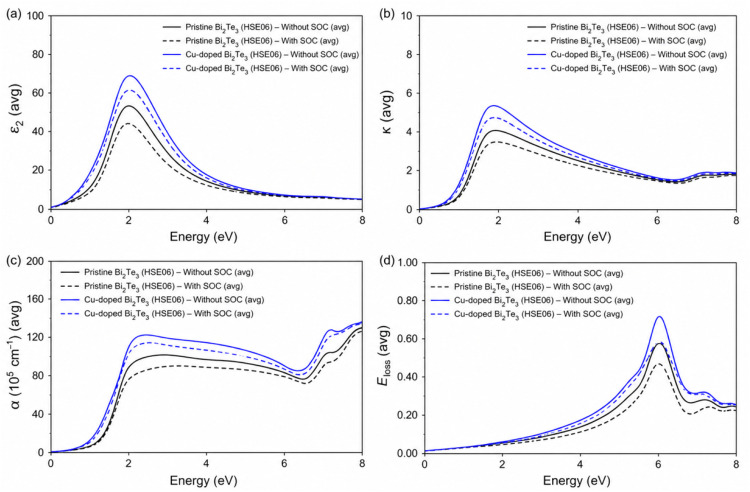
Polarization-averaged optical properties of pristine and Cu-doped Bi_2_Te_3_ calculated using the HSE06 functional without and with spin–orbit coupling: (a) imaginary dielectric function, *ε*_2_(*ω*), (b) extinction coefficient, *κ*(*ω*), (c) absorption coefficient, *α*(*ω*), and (d) energy-loss function, *E*_loss_(*ω*). Cu doping enhances the optical response, particularly near the main interband transition around 2 eV, while SOC slightly reduces peak intensities and broadens the spectra due to relativistic band splitting and orbital rehybridisation.

Fig. 9 shows the polarization-averaged optical response of pristine and Cu-doped Bi_2_Te_3_ obtained using HSE06, with and without SOC. The spectra include the imaginary dielectric function, *ε*_2_(*ω*), extinction coefficient, *κ*(*ω*), absorption coefficient, *α*(*ω*), and energy-loss function, *E*_loss_(*ω*), over the 0–8 eV range.

The imaginary dielectric function in Fig. 9a exhibits a dominant peak around 2.0 eV for all systems, indicating intense interband transitions from Te-p/Bi-p valence states to Bi-p dominated conduction states. Cu doping significantly enhances *ε*_2_(*ω*), with the Cu-doped system showing the strongest peak intensity. This indicates that Cu incorporation increases the optical transition probability by modifying the near-band-edge electronic states. SOC slightly reduces the peak intensity and broadens the spectral profile, which is consistent with SOC-induced band splitting and redistribution of oscillator strength in Bi_2_Te_3_-type compounds.

The extinction coefficient, *κ*(*ω*), in Fig. 9b follows the same trend as *ε*_2_(*ω*). A pronounced maximum is observed near 1.8–2.0 eV, followed by a gradual decrease toward higher photon energies. The Cu-doped systems again show larger *κ* values than pristine Bi_2_Te_3_, confirming stronger light attenuation. The SOC-included spectra show slightly lower maxima, suggesting that SOC redistributes the transition strength rather than introducing a new dominant optical channel.

The absorption coefficient in Fig. 9c reaches values of the order of 10^5^ cm^−1^, confirming strong absorption from the visible to near-ultraviolet region. This magnitude is consistent with previous optical studies of Bi_2_Te_3_ thin films and related layered chalcogenides, which have reported strong absorption over a broad spectral range.^50–52^ Cu-doped Bi_2_Te_3_ shows higher absorption than the pristine system over most of the 2–6 eV range, implying that Cu-induced charge redistribution strengthens optically active electronic transitions. This agrees with doped Bi_2_Te_3_ studies where dopant-induced changes in the electronic structure were found to enhance optical absorption in the visible and near-UV regions.^53^

The energy-loss spectra in Fig. 9d show a strong plasmon-related maximum around 6.0–6.3 eV. The Cu-doped system without SOC gives the highest loss peak, while SOC slightly suppresses and broadens the response. The position of the main loss peak is close to values reported for Bi_2_Te_3_-based systems, where plasmonic features are commonly observed in the higher-energy optical region^54^ (see Table 6). The relatively small SOC-induced shift suggests that SOC mainly modifies the fine structure of the interband transitions, while the overall collective excitation energy remains governed by the Bi–Te host framework.

**Table 6:** Comparison of reported optical properties of Bi_2_Te_3_ and doped Bi_2_Te_3_ systems from experimental and theoretical studies, including dielectric response, absorption coefficient, extinction coefficient, and energy-loss features, alongside the present HSE06 results. The table highlights the influence of Cu doping on enhancing optical transition intensity and confirms that spin–orbit coupling (SOC) primarily refines spectral features through band splitting and redistribution of oscillator strength without significantly altering the overall optical profile

System/method	Reported optical behavior	Comparison with present HSE06 results
Bi_2_Te_3_ epilayers, ellipsometry and reflectivity	Optical constants measured from far-IR to UV; strong anisotropic optical response reported	Supports the strong optical response and layered anisotropy observed here^51,52^
Bi_2_Te_3_ thin film, first-principles optical study	Strong absorption from near-IR to UV.	Consistent with *α*(*ω*) reaching ∼10^5^ cm^−1^ in this work^53^
Doped Bi_2_Te_3_, DFT-based optical study	Doping enhances absorption in visible and near-UV regions	Agrees with the stronger absorption obtained for Cu-doped Bi_2_Te_3_^54^
Bi_2_Te_3_ optical constants/topological-insulator optics	High optical polarizability and plasmonic response reported over visible-near-IR regions	Supports the observed high extinction and energy-loss features^55^
First-principles dielectric study of Bi_2_Te_3_	Layered Bi_2_Te_3_ shows anisotropic dielectric response	Consistent with the direction-dependent spectra and averaged optical trends^56^

Overall, the optical spectra show that Cu doping enhances the dielectric response, extinction coefficient and absorption coefficient without destroying the intrinsic optical character of Bi_2_Te_3_. SOC produces a more refined description by reducing peak intensities, broadening spectral features and slightly shifting the energy-loss maximum. These results are consistent with the electronic-structure analysis: Cu redistributes charge and increases the density of optically active states, while SOC corrects the band ordering and orbital hybridisation associated with heavy Bi and Te atoms.

#### 3.1.7. Optical anisotropy: birefringence and dichroism

**Fig. 10:**
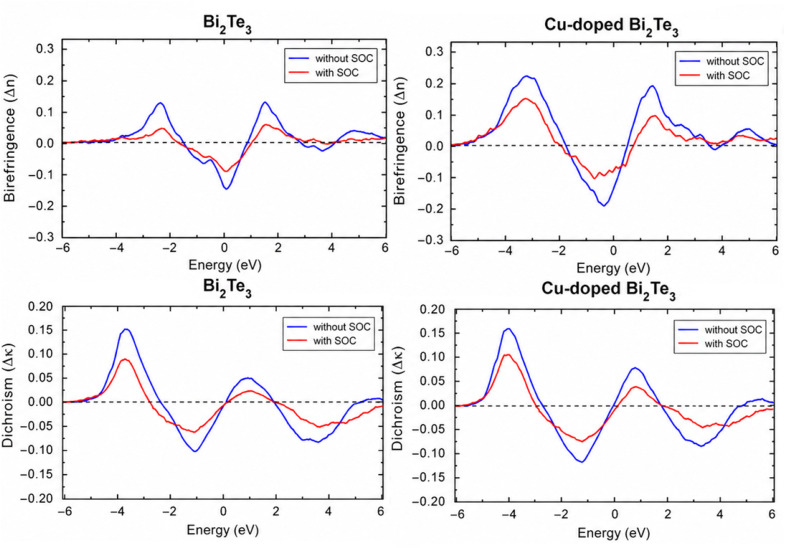
Birefringence (Δ*n*) and dichroism (Δ*κ*) spectra of pristine and Cu-doped Bi_2_Te_3_ calculated using HSE06, with and without SOC. Solid and dashed lines represent without and with SOC, respectively. Pristine is shown in black and Cu-doped in blue, highlighting doping-induced changes and weak SOC effects.

The anisotropic optical response of pristine and Cu-doped Bi_2_Te_3_ was further examined through the calculated birefringence (Δ*n*) and dichroism (Δ*κ*), as shown in Fig. 10. These quantities provide a sensitive probe of directional variations in the complex dielectric function and are therefore directly linked to the underlying electronic structure and orbital hybridisation.

For pristine Bi_2_Te_3_, the birefringence spectrum exhibits a characteristic oscillatory behaviour over the investigated energy range. Distinct extrema are observed in both the valence- and conduction-band regions, with a sign reversal occurring near the Fermi level. This behaviour reflects the intrinsic anisotropy of the layered rhombohedral structure, where strong in-plane bonding within quintuple layers contrasts with weaker interlayer interactions. As a result, the refractive index differs along crystallographic directions, giving rise to measurable birefringence. The magnitude of Δ*n* remains moderate, indicating that although the system is anisotropic, the optical response is not strongly polarised in any single direction.

The inclusion of spin–orbit coupling leads to a noticeable reduction in the magnitude of birefringence across the entire energy range. This effect can be attributed to SOC-induced band splitting and enhanced mixing of orbital characters, particularly involving Bi-*p* states. The redistribution of electronic states reduces the directional contrast in optical transitions, thereby diminishing the anisotropy. This observation is consistent with the known role of SOC in heavy-element systems, where relativistic effects tend to smooth out anisotropic features in the electronic and optical response.

A more pronounced anisotropic behaviour is observed in Cu-doped Bi_2_Te_3_. The magnitude of Δ*n* increases significantly compared to the pristine system, especially in the energy regions corresponding to strong interband transitions. This enhancement suggests that Cu incorporation introduces additional asymmetry in the electronic polarizability. From the electronic-structure perspective, this can be understood in terms of hybridisation between Cu-d states and the host Bi–Te p orbitals, leading to a redistribution of charge density and direction-dependent transition probabilities. Despite this enhancement, the inclusion of SOC again reduces the overall birefringence, indicating that relativistic effects counterbalance the anisotropy introduced by doping.

The dichroism spectra (Δ*κ*) show trends similar to those of birefringence but provide insight into anisotropic absorption processes. In pristine Bi_2_Te_3_, Δ*κ* exhibits prominent features in the valence-band region, associated with direction-dependent interband transitions. The inclusion of SOC leads to a reduction in peak intensities, particularly at lower energies, reflecting a more uniform distribution of absorption across different polarisation directions.

In the Cu-doped system, dichroism is enhanced relative to pristine Bi_2_Te_3_, with larger amplitude variations observed across the energy spectrum. This indicates that Cu doping increases the anisotropy of optical absorption, likely due to the introduction of additional electronic states that participate differently in transitions along distinct crystallographic directions. As observed for birefringence, SOC reduces the magnitude of Δ*κ* but does not eliminate the enhanced anisotropic response induced by Cu.

Overall, these results highlight a clear interplay between Cu doping and spin–orbit coupling in determining the optical anisotropy of Bi_2_Te_3_. While Cu incorporation enhances directional optical response through charge redistribution and orbital hybridisation, SOC acts to moderate this effect by redistributing electronic states and reducing directional contrasts. This balance between doping-induced anisotropy and SOC-driven homogenisation is an important factor in tailoring the optical properties of Bi_2_Te_3_-based materials.

The present findings are in good agreement with earlier studies on layered chalcogenides, where strong structural anisotropy leads to direction-dependent optical properties, and SOC has been shown to play a critical role in shaping the dielectric response of heavy-element systems. In particular, previous theoretical and experimental investigations have reported that Bi_2_Te_3_ exhibits moderate birefringence and anisotropic absorption, both of which are sensitive to band structure modifications and external perturbations such as doping.^55,57,58^ The enhancement of optical anisotropy upon doping, coupled with the moderating effect of SOC, therefore provides a consistent and physically meaningful picture that complements the electronic and thermoelectric analyses presented in this work.

#### 3.1.8. Selective light modulation efficiency (SLME)

**Fig. 11:**
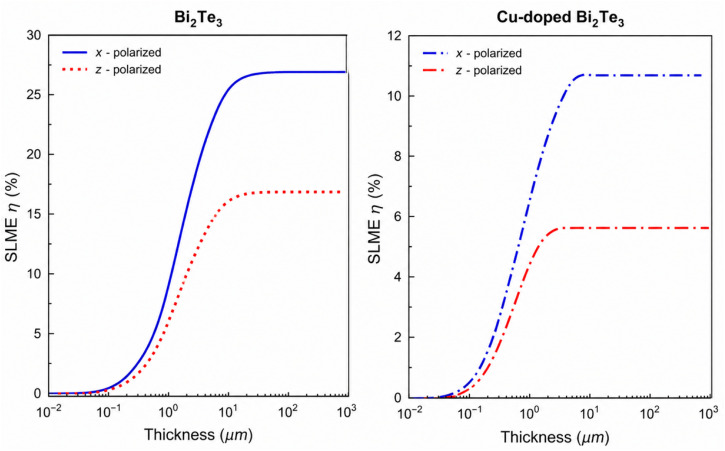
Thickness-dependent selective light modulation efficiency (SLME) of pristine and Cu-doped Bi_2_Te_3_ for in-plane (*x*) and out-of-plane (*z*) polarizations. Pristine Bi_2_Te_3_ exhibits higher SLME values and stronger anisotropy, while Cu doping reduces the overall efficiency but preserves the characteristic thickness dependence and directional behavior.

The thickness-dependent selective light modulation efficiency (SLME) of pristine and Cu-doped Bi_2_Te_3_ is presented in Fig. 11 for two representative polarization directions (in-plane, *x*, and out-of-plane, *z*). SLME provides a more realistic assessment of photovoltaic and optoelectronic performance than conventional absorption metrics, as it incorporates radiative recombination losses and thickness-dependent absorption.

For pristine Bi_2_Te_3_, the SLME increases rapidly with film thickness in the sub-micrometre to micrometre regime, followed by a gradual saturation at larger thicknesses. The in-plane (*x*-polarized) response reaches a maximum of approximately ∼27%, while the out-of-plane (*z*-polarized) response saturates at a lower value of ∼16–17%. This pronounced anisotropy reflects the layered crystal structure of Bi_2_Te_3_, where stronger in-plane optical transitions and carrier transport lead to enhanced light-harvesting efficiency compared to the out-of-plane direction. The rapid rise in SLME at low thickness indicates strong absorption coefficients, consistent with the optical spectra discussed earlier.

In contrast, Cu-doped Bi_2_Te_3_ exhibits a reduced overall SLME compared to the pristine system, with maximum values of ∼10–11% for *x*-polarization and ∼5–6% for *z*-polarization. The reduction in SLME upon Cu incorporation can be attributed to changes in the electronic structure that affect the balance between absorption strength and recombination losses. While Cu doping enhances certain optical transitions and increases the density of states near the Fermi level, it may also introduce additional scattering channels or modify carrier lifetimes, leading to reduced effective photovoltaic efficiency.

Despite the reduction in magnitude, the Cu-doped system preserves the same qualitative thickness dependence and anisotropic behaviour observed in pristine Bi_2_Te_3_. The SLME curves for both polarizations exhibit a similar sigmoidal profile, with a steep increase at intermediate thickness followed by saturation. This suggests that the fundamental optical absorption characteristics remain intact, and that Cu primarily acts as a perturbative modifier rather than fundamentally altering the light–matter interaction mechanism.

The anisotropy between *x*- and *z*-polarized responses remains significant in the doped system, reinforcing the role of structural layering in governing optical performance. The consistently higher SLME for in-plane polarization highlights the importance of orientation in potential device applications, particularly for thin-film configurations where directional properties can be exploited.

From a broader perspective, the SLME values obtained for pristine Bi_2_Te_3_ are comparable to those reported for narrow-gap semiconductors with strong absorption in the visible region. Previous studies have shown that materials with high absorption coefficients and moderate band gaps can achieve SLME values exceeding 20% under optimal conditions. The present results place Bi_2_Te_3_ within this category, although its primary application remains thermoelectric rather than photovoltaic. The reduced SLME in the Cu-doped system suggests a trade-off between electronic tuning for thermoelectric enhancement and optical efficiency, which is an important consideration for multifunctional material design.

Overall, the SLME analysis complements the optical and electronic results, demonstrating that while Cu doping enhances certain aspects of electronic structure relevant to thermoelectric performance, it does not necessarily improve light-harvesting efficiency. The interplay between absorption, recombination and anisotropy must therefore be carefully balanced when targeting applications that involve both optical and electronic functionalities.

## 4. Conclusions

In this work, a comprehensive first-principles investigation of pristine and Cu-doped Bi_2_Te_3_ has been carried out using the HSE06 hybrid functional with explicit inclusion of spin–orbit coupling. The results demonstrate that Cu incorporation induces a slight lattice expansion while preserving structural, dynamical, thermal, and mechanical stability. Phonon and elastic analyses confirm that the doped system remains robust, with moderate lattice softening that is favourable for phonon scattering.

The electronic structure is found to be strongly governed by spin–orbit coupling, which is essential for accurately reproducing the narrow band gap and band-edge features. Cu doping does not significantly alter the fundamental band gap but modifies the near-Fermi-level dispersion and density of states, promoting improved carrier transport. As a result, the Seebeck coefficient is enhanced while maintaining reasonable electrical conductivity, leading to an overall improvement in the power factor.

Optical calculations reveal that Cu incorporation strengthens the dielectric response and absorption, while spin–orbit coupling refines spectral features through band splitting. The combined effect of dopant-induced charge redistribution and relativistic interactions therefore provides a balanced route to tune both electronic and optical properties.

Overall, this study highlights Cu doping as an effective strategy for optimising Bi_2_Te_3_-based thermoelectric materials, and underscores the importance of incorporating spin–orbit coupling for a physically reliable description of their properties.

## Conflicts of interest

The authors have no conflicts of interest to declare.

## Data Availability

Computational input/output files are available from the corresponding author upon reasonable request.
